# Probiotic Supplementation and Micronutrient Status in Healthy Subjects: A Systematic Review of Clinical Trials

**DOI:** 10.3390/nu13093001

**Published:** 2021-08-28

**Authors:** Bahareh Barkhidarian, Lucas Roldos, Michèle M. Iskandar, Ahmad Saedisomeolia, Stan Kubow

**Affiliations:** 1 Department of Cellular and Molecular Nutrition, School of Nutritional Sciences and Dietetics, Tehran University of Medical Sciences, Keshavarz Blvd., Tehran 1471613151, Iran; bahar.darian@gmail.com; 2 School of Human Nutrition, McGill University, 21111 Lakeshore, Sainte-Anne-de-Bellevue, QC H9X 3V9, Canada; lucas.roldos@mail.mcgill.ca (L.R.); michele.iskandar@mail.mcgill.ca (M.M.I.)

**Keywords:** probiotics, micronutrients, B vitamins, vitamin A, vitamin D, vitamin E, folate, calcium, iron, zinc

## Abstract

Micronutrient deficiencies are a worldwide public health concern. Emerging evidence supports the ability of probiotics to enhance micronutrient status, which could aid in the prevention of non-communicable disease-associated malnutrition. This systematic review evaluated evidence of the efficacy of probiotic supplementation to improve micronutrient status in healthy subjects. The authors searched for published English language peer-reviewed journal articles in PubMed, Scopus, Embase, and Google Scholar databases from inception to July 2020 using Preferred Reporting Items for Systematic Reviews and Meta-Analyses (PRISMA) guidelines. The quality of eligible studies was assessed using the Revised Cochrane Risk-of-Bias tool (RoB)2 and Risk of Bias in Non-Randomized Studies of Interventions tool (ROBINS-I tool). Fourteen original studies out of 2790 met the inclusion criteria. The results indicated that, despite varying degrees of efficacy, the intake of certain probiotics in healthy subjects was associated with a positive impact on the status of certain micronutrients (vitamin B12, calcium, folate, iron and zinc). A limitation was that studies were widely heterogeneous in terms of participant age, probiotic strain, species, dosage, intervention duration, and form of administration. Additional clinical trials are warranted to determine the most effective strains of probiotics, doses and durations of interventions.

## 1. Introduction

Micronutrients are organic or inorganic food compounds that are not used for energy but are essential for the maintenance of health and include vitamins and minerals. Inadequate intake of micronutrients can cause specific micronutrient deficiency diseases that are a major public health concern affecting two billion people worldwide [1]. Micronutrient deficiencies can aggravate infection and chronic diseases, such as osteoporosis, hypothyroidism, colorectal cancer, and cardiovascular diseases, and consequently have a great impact on morbidity, mortality and quality of life [1,2]. A variety of interventions, such as large-scale food fortification, have been used to improve micronutrient status on a population level to decrease disease burden. Despite such public health programs, micronutrient deficiencies in terms of iron, vitamin A and zinc continue to be major global health issues [3].

A role for microorganisms in the modulation of host micronutrient status has been suggested because food-associated transiting microbes, probiotic bacteria and commensal bacteria in the human gut are known to synthesize vitamins [4] and affect host nutrient absorption [5]. According to the World Health Organization, probiotics are defined as “live microorganisms which when administered in adequate amounts confer a health benefit on the host” [6]. The most common probiotics are the genera *Lactobacillus* and *Bifidobacterium* [7], which are normally found in fermented foods and dairy products [8]. Probiotics can enter the host via food or supplements. Despite obstacles such as gastric acidity and bile acids that impede the viability of administered probiotic microorganisms, probiotics can survive in sufficient numbers [9] to colonize colonic regions to provide health benefits, including vitamin production [10,11]. Consequently, probiotics could be a novel approach to enhance micronutrient status and thereby combat non-communicable disease-associated malnutrition. An expert workshop on the influence of microbiota and probiotics and/or prebiotics on malnutrition held by the International Scientific Association for Probiotics and Prebiotics (ISAPP) has called for more clinical studies assessing the use of probiotics to combat malnutrition [12].

Depending on the micronutrient, there are a number of possible mechanisms by which probiotics can optimize the intestinal milieu for better nutrient absorption. The proposed mechanisms include: (a) decreased pH via increased intraluminal lactic acid production [13,14,15]; (b) modified hormone levels [16]; (c) beneficial alterations of gut microbiota populations [17]; and (d) inhibition of pathogenic bacterial adhesion to the intestinal epithelial cell surface that consequently reduces competition with the host for available nutrients [18]. Additionally, some species of bacteria (Lactobacillus and Bifidobacterium) can produce vitamins (particularly, B vitamins and vitamin K) [19,20,21,22,23]. However, the mechanisms underlying possible probiotic-induced improvements in micronutrient status still require more investigation.

The ability of probiotics to increase micronutrient absorption [13,24,25] and status [16,26,27,28,29] has been examined extensively in both animal models and clinical studies. Improvements in nutrient status following administration of certain probiotics have been noted regarding B-group vitamins (folate and B12) [30,31,32], and minerals (iron and calcium) [29,33,34,35]. To date, mixed results of probiotic supplementation have been reported from clinical trials across the different micronutrients. This systematic review was conducted to present an updated comprehensive evaluation of existing evidence regarding the efficacy of probiotic supplementation to improve micronutrient status in healthy humans and to provide directions for future research.

## 2. Materials and Methods

### 2.1. Literature Search Strategy

The present systematic review followed Preferred Reporting Items for Systematic Reviews (PRISMA) guidelines [36] in all steps. The literature search was conducted using the electronic bibliographic databases PUBMED, SCOPUS, and Embase from inception to July 2020 in consultation with a research librarian. In addition, Google Scholar was spot searched to find relevant studies that may have been published in journals that are not indexed in bibliographic databases [37,38]. The inclusion criteria considered original intervention studies published in English that assessed the relationship between probiotic intake and micronutrient profiles. Case reports, book chapters, review papers, editorials and animal model or cell culture studies were excluded. The search pattern was employed in PubMed, but the search strategy was modified for individual databases. The search strategies are available in Appendix A. Studies were selected according to the PICOS design (PICOS: patients, intervention, comparator, outcome and study design): the population included “healthy adults and children”; intervention included “probiotic supplementation”; comparison included “adequate control or placebo”; outcomes included “micronutrient status, level, or concentration”; study design included “human clinical trials”. The reference lists of all included studies were reviewed to find other potentially eligible articles.

**Figure 1:**
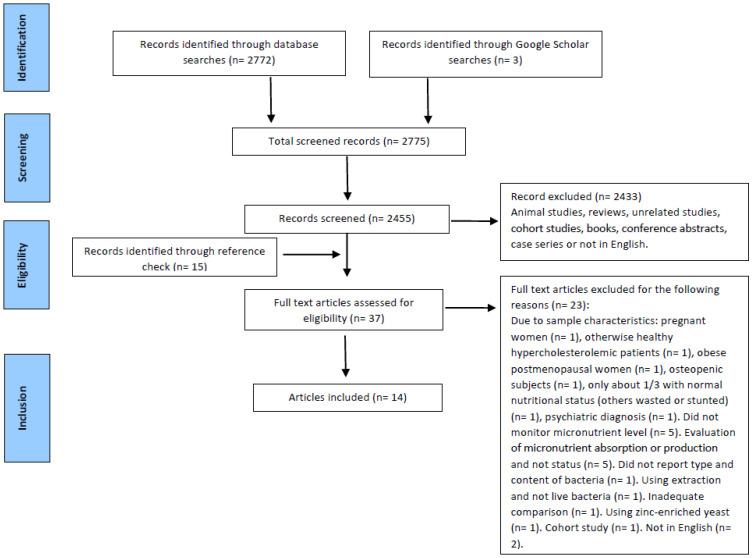
Search and inclusion process flow chart of studies to include in this systematic review of the association between probiotic supplementation and micronutrient status in healthy subjects.

The search strategy is summarized in Figure 1.

### 2.2. Eligibility Criteria and Study Selection

The results of database searches were first imported to EndNote software (version X8, for Windows, Thomson Reuters, Philadelphia, PA, USA). Duplicate studies were removed before screening of the remaining studies. Two authors (B.B. and L.R.) independently screened each article by title, then abstract, and then full text, for inclusion. Discrepancies were resolved through discussion. The screening of titles and abstracts were completed according to the following inclusion criteria: (1) clinical trials with healthy human subjects; (2) intervention with probiotic supplementation; and (3) reporting outcomes related to micronutrient status including vitamins A, C, D, E, K, B vitamin group (thiamin (B1), riboflavin (B2), niacin (B3), pyridoxine (B6), folic acid, cobalamin (B12), pantothenic acid, biotin, choline), carotenoids and minerals (calcium, iron, zinc, magnesium, manganese, molybdenum, selenium, potassium, chromium, copper, sodium, phosphorus, iodine, fluoride, chloride). Only English language articles were included in the present review.

### 2.3. Data Abstraction

Extracted data included: author’s name, year, country of study, study design, sample size and characteristics, treatment groups and probiotic strains/species, form of probiotic administration, frequency and duration, and the outcomes.

### 2.4. Quality Assessment

The methodological quality of each included study was independently assessed by two authors (BB and LR) using the Revised Cochrane Risk-of-Bias tool (RoB)2 [39,40] and the Risk Of Bias In Non-Randomized Studies of Interventions tool (ROBINS-I tool) [41]. For parallel and cross-over randomized controlled trials (RCTs), the following methodological domains were considered: (a) randomization process; (b) deviation from the intended interventions; (c) missing outcome data; (d) measurement of the outcome; and (e) selection of the reported results. For each criterion, bias was assessed as a judgment (expressed as “high risk of bias”, “low risk of bias”, or “some concerns”). For non-randomized clinical trials, the assessed methodological domains were as follows: bias due to confounding; bias in selection of participants into the study; bias in classification of interventions; bias due to deviations from intended interventions; bias due to missing data; bias in measurement of outcomes; and bias in selection of the reported result. Discrepancies between two authors were resolved through discussion.

## 3. Results and Discussion

### 3.1. Study Selection

Figure 1 depicts the results of the literature search. The initial literature search identified 2772 abstracts. There were also three more articles found on Google Scholar. After removing duplicates (n = 320) and screening studies by title and abstract, 22 human clinical trials were retrieved for full-text review. After evaluation of reference lists to identify other relevant studies and elimination of articles that did not meet inclusion criteria, a total of 14 articles were included for this review (Supplementary Table S1).

This section may be divided by subheadings. It should provide a concise and precise description of the experimental results, their interpretation, as well as the experimental conclusions that can be drawn.

### 3.2. Quality Assessment

The evaluated studies differed in terms of methodological quality. Supplementary Figures S1 and S2 describe the risk of bias for the included studies. Only one of the fourteen included studies had a low risk of bias. The rest of the parallel and cross-over double-blind randomized clinical trials revealed some concerns in their overall risk of bias results. Key sources of bias in these studies were: (1) randomization process; and (2) deviation from the intended interventions. All three non-randomized trials demonstrated a serious risk of bias. In non-randomized clinical trials, bias due to confounding was the main concern. We believe that more well-designed, double-blind, randomized studies are needed in this area. Moreover, authors of such studies should properly describe the randomization sequence and concealment as well as the type of blinding implemented.

### 3.3. Characteristics of the Included Studies

All fourteen clinical trials were published in journals between the years 2000 and 2020. Studies were conducted in Austria [42,43,44], Indonesia [45,46], USA [47], India [48], Sweden [49], Italy [50], Egypt [51], Finland [16], Brazil [28], Serbia [52], and one was a multinational study [32]. Study design across the 14 included studies varied. Four studies were randomized double-blind placebo-controlled trials [45,46,49,50], two others used a cross-over design [16,48], one was an open-label, randomized, multicenter study [32], and four randomized trials did not report blinding [43,44,47,51]. Three studies were nonrandomized trials [28,42,52]. Sample sizes for all fourteen included studies ranged from 12 to 494. Four studies had a sample size of less than thirty participants [16,42,47,51,52] and two studies with the largest sample sizes (109 and 494) were conducted on healthy children [28,46].

Beyond being healthy, studies varied in participant characteristics. Five studies included children [28,45,46,50,51], six included adults [42,43,44,47,49,52] and three included older subjects [16,32,48]. One study included non-anemic, iron deficient athletes [49] and another included vegan subjects [47]. Five studies included females only [16,40,43,49,52] and nine studies included both sexes [28,32,42,45,46,47,48,50,51]. The study characteristics are listed in Supplementary Table S1.

Different forms of probiotic administration were used across the included studies. Studies conducted using prebiotics only were excluded. One study evaluated the effect of a synbiotic [50] and thirteen evaluated the effect of probiotics [16,28,32,42,43,44,45,46,47,48,49,51,52]. A synbiotic is defined by ISAPP as “a mixture comprising live microorganisms and substrate(s) selectively utilized by host microorganisms that confers a health benefit on the host” [53]. Some studies used probiotic or synbiotic supplements in capsule [32,47,49,52], tablet [50] or powder [45] forms, while others used fortified/fermented milk [16,28,46,48] or a probiotic yogurt [42,43,44,51]. Strains or species of bacteria also differed among studies. Seven studies used multi-strain probiotics or synbiotics [32,42,43,44,47,48,50], three studies used *L. plantarum* [45,49,52], two used *L. acidophilus* [28,51], and one used *L*. *helveticus* [16]. In addition, one study used both *L. rueteri* and *L. casei* in two separate groups [46]. Duration of intervention in parallel RCTs and nonrandomized clinical trials varied widely from 7 days to 6 months. The characteristics and doses of the probiotics used by the included studies are summarized in Supplementary Table S1.

#### 3.3.1. Probiotic Supplementation and Fat-Soluble Vitamin and Carotenoid Status

Two clinical studies involving healthy subjects assessed the effect of probiotics on fat-soluble vitamin and carotenoid status [43,50]. One randomized double-blind clinical trial supplemented forty 14–18 year-old pediatric participants with a synbiotic (probiotic and prebiotic combination) tablet (*L. plantarum, L. acidophilus, B. infantis, B. lactis* (45 × 10^9^ CFU) and fructo-oligosaccharides (FOS)) for 10 weeks [50]. The overall result of the risk of bias assessment for the study revealed concerns, as there was no explicit explanation of the blinding performed and the vitamin A and D measures were secondary outcomes [43,50]. The study results indicated no difference in vitamin A status between the synbiotic and placebo groups. Similarly, no benefit in vitamin A status was observed in pregnant women receiving synbiotic powdered fortified milk containing *Bifidobacterium animalis* subsp. *lactis* HN019 and inulin [54]. In the above mentioned pediatric trial [50], an increase in vitamin D status was indicated with the synbiotic treatment; however, the p values were not reported. In concert with the latter study, an increase in vitamin D status as assessed by serum 25-hydroxy vitamin D3 was observed after *L. reuteri* NCIMB30242 consumption [14] and following multi-strain probiotic consumption in two studies with bariatric surgery patients [30,55]. In contrast, the synbiotic intervention trial in pregnant women using *Bifidobacterium animalis lactis* HN019 and inulin did not show an improvement in vitamin D status [54]. The inconsistent results may be due to differences between study populations, and the use of different strains (*L. reuteri* [14], *B.* *animalis* [54], and multi-strain probiotics) [30,55], doses and durations of intervention. Therefore, larger well-designed RCTs are needed to reach a consensus on the effects of probiotic supplementation on vitamin D status. The other study was a randomized trial that supplemented 32 healthy women with conventional yogurt containing *S. thermophilus*, 3.9 × 10^7^ CFU/g, *L. bulgaricus*, 6.4 × 10^7^ CFU/g or probiotic yogurt containing *S. thermophilus* 2 × 10^8^ CFU/g and *L. bulgaricus* 10^7^ CFU/g, enriched with a probiotic culture of *L. paracasei* (subsp. *Paracasei*) (*L. casei* DN-114 001, 3.6 × 10^8^ CFU/g) for one month [43]. Participants received 100 g of their assigned yogurt in the first two weeks and 200 g in the second two weeks. The overall result of the risk of bias assessment for the above study revealed some concerns, as there was no explicit explanation of the randomization process and blinding [43,50]. The results indicated that the plasma levels of vitamin E, lycopene and zeaxanthin decreased significantly in both the probiotic and control groups. Interestingly, the plasma concentrations of lutein and β-carotene decreased significantly in the probiotic group only. While these results may suggest that this yogurt and specific probiotics may reduce vitamin E and carotenoid status, it is important to note that the probiotic and conventional yogurt differed in terms of starter cultures dosage and macronutrient composition. The probiotic yogurt had less fat (1.6 vs. 3.6 g/100 g), protein (2.8 vs. 3.4 g/100 g), and more carbohydrates (14.2 vs. 3.9 g/100 g) than the conventional yogurt [43]. Despite these differences, the dietary intake of total energy and tocopherol equivalents remained stable during the study in both groups [43]. Another intervention trial with 127 otherwise healthy hypercholesterolemic subjects revealed similar results whereby 9 weeks of *L. reuteri* NCIMB30242 supplementation did not influence the status of vitamin A, vitamin E and β-carotene [14]. To date, data concerning the effects of probiotic intake on vitamin A, vitamin E and carotenoid status are scarce, and larger well-designed RCTs are needed.

#### 3.3.2. Probiotic Supplementation and Vitamin B Group Status

Two clinical studies evaluated the effect of probiotics on vitamin B1, B2 and B6 status [42,44]. One non-randomized trial was conducted on both healthy men and women [42]. This two-week study compared the effect of thermally inactivated cultured yogurt versus yogurt without heat treatment containing (*S. thermophilus* plus *L. acidophilus* and *L. casei* (*rhamnosus*) SGG (LGG) at 5 × 10^7^ CFU/g) [42]. Although, the intervention showed no effect on vitamin B1, B2, and B6 status, this trial was deemed to have a serious risk of bias as the authors did not mention the dosage of the yogurt starter culture. The second study was a randomized trial that investigated the effect of one month probiotic or traditional yogurt intake on the status of vitamins B1, B2, and B6 in healthy women [44]. The control yogurt contained the starter cultures *S. thermophilus*, 3.9 × 10^7^ CFU/g and *L. bulgaricus*, 6.4 × 10^7^ CFU/g. The probiotic yogurt contained starter culture enriched with *L. paracasei* (subsp. *Paracasei*) (*L. casei* DN-114 001, 3.6 × 10^8^ CFU/g). Participants received 100 g of each yogurt in the first two weeks and 200 g in the second two weeks. No significant difference was observed in vitamin B1, B2 and B6 status between probiotic and control groups [44]. This randomized trial showed some concerns in the risk of bias assessment as the vitamin B1 and B2 content of the conventional and probiotic yogurts were different and the average intake of vitamins B1 and B2 changed significantly within both groups during the trial. Therefore, the changes observed in the plasma levels of these vitamins within each group could have stemmed from yogurt intake per se [44]. The initial plasma and urine concentrations of all investigated B vitamins were within the normal range in both of the above studies. *L. casei*, which was used in both studies, is among strains that produce thiamine, but the vitamin is only released upon lysis of the bacterial cell [42]. Since *L. casei* survives the acidity of the stomach and can colonize the colon [56], there is a possibility that the amount of thiamin (vitamin B1) available to the upper gastrointestinal regions is not very high [42]. Previous studies have shown that some strains of lactic acid bacteria (LAB) or *Bifidobacteria* have the ability to produce vitamins B1 and B2. *B. longum* and *B. bifidum* are among strains that have been shown to be able to produce vitamin B1 [57]. *L. fermentum* has the ability to produce vitamin B2 [58] and *L. rhamnosus* GG is able to produce both vitamins B1 and B2 [22]. Therefore, these strains may be good choices for future studies that investigate the effect of probiotics on thiamin or riboflavin status. Overall, larger well-designed RCTs with comprehensive dietary intake evaluation and better control of vitamin B1 and B2 intake, accompanied by use of appropriate probiotic strains, may provide better insights.

Three studies evaluated the effect of probiotic consumption on folic acid and vitamin B12 levels [32,47,51]. A randomized nutritional supplementation trial included 24 healthy children (8/24 deficient in vitamin B12, 5/24 deficient in folate) and evaluated the effect of viable *L. acidophilus* (La1) 5 × 10^9^ CFU in 2 cups of yogurt daily on the plasma levels of vitamin B12 and folate [51]. This trial had some concerns regarding risk of bias as there was no explicit description of the randomization process, concealment and blinding. The results showed that probiotics improved plasma levels of vitamin B12 and folate along with significant decreases in urinary excretion of both methylmalonic acid (MMA) and total homocysteine (Hcy). These latter biomarkers are pertinent as low blood levels of vitamin B12 do not always represent vitamin B12 deficiency, and insufficient B12 intake is consistently associated with accumulation of Hcy and MMA [59]. For similar reasons, low folate status is assessed by elevated blood levels of Hcy [60]. A randomized open label multi-center trial determined the impact of a personalized diet with or without VSL#3 on vitamin B12, folate and Hcy levels in healthy older patients [32]. The VSL#3 supplement contains 112 billion lyophilized bacteria consisting of the following strains: *B. infantis* DSM 24,737, *B. longum* DSM 24,736, *B. breve* DSM 24,732, *L. acidophilus* DSM 24,735, *L. delbrückii* subsp. *bulgaricus* DSM 24,734, *L. paracasei* DSM 24,733, *L. plantarum* DSM 24,730 and *S. thermophilus* DSM 24,731, in a specific ratio that was not reported. This trial revealed some concerns in the risk of bias assessment as it was an open-label trial with no blinding or concealment process. The results demonstrated that probiotics improved the levels of plasma vitamin B12 and folate and significantly decreased plasma Hcy levels.

Some mechanisms have been described for the positive effects of probiotics on folate levels. The production of folate in the gastrointestinal tract by some bacterial species could be an explanation [23]. Among the bacteria that improved folate levels, *L. acidophilus* is not able to produce folate [61], although, *L. plantarum* [23,62], *S. thermophilus* [20], *B. infantis* [10,61,63], *B. breve* [10,61] and *B. longum* [10,61], which are present in VSL#3 can generate folate. Another strain of importance is *B. adolescentis* DSM 18,350 as it has been shown to produce folate [10], increase fecal concentrations of folate [64], and improve folate status in rats [11]. Experimental studies suggest that certain strains of *Bifidobacteria* may also be used to increase folate uptake [65]. Thus, several of the above strains appear to have potential to improve folate status, but further clinical trials are needed to explore optimal intervention strategies.

There is also evidence that probiotics may exert their beneficial effect on the host through changes in gut microbiome composition [66,67]. Previous studies have shown that a mixture of probiotic strains can change the gut microbiota of the elderly and indicate increased fecal *Bifidobacterium* [68]; however, in the above study by Valentini et al. 2015 [32] showing improvement in vitamin B12 and folate status in older subjects, no significant change was observed in the fecal abundances of *Bifidobacterium* spp. Interestingly, subgroup analysis showed that all participants with low-grade inflammation demonstrated increased bifidobacteria populations after VSL#3 intervention with a mean increase that was significantly higher when compared to diet alone. In contrast, the above findings were not noted in participants without low-grade inflammation. Apart from the production of vitamin B12 by some probiotic bacteria, e.g., *L. plantarum* [69], probiotics may enhance vitamin B12 status by changing the gut microbiome composition [70] and decreasing the number of intestinal bacteria that catabolize B12 [31]. Despite these purported mechanisms, there is one study on vegan subjects that reports no change in vitamin B12 levels after receiving probiotics [47]. A randomized trial examined folate and vitamin B12 status of vegan subjects following supplementation of a sublingual cyanocobalamin tablet, nutritional yeast, and one of two selected probiotic supplements (Probiotic Formula containing the five bacteria: *L. plantarum, L. salivarius, L. acidophilus, B. bifidus* and *Bacillus subtilis* and Flora Food containing *L. salivarius* and *L. plantarum*) [47]. This study has some concerns in the bias assessment as there was no explicit description of the randomization process; moreover, the trial was not blinded. Neither probiotic decreased urinary MMA whereas both the cyanocobalamin tablet and nutritional yeast supplements decreased urinary MMA for most participants in both groups. These contradictory results could be linked to the dietary habits of the participants, as this will influence their initial micronutrient status that may affect the efficacy of the probiotic intervention. Since cobalamin is not found in plant foods, vegans are more likely to be deficient in vitamin B12. In the vegan study, 23 out of 24 participants had elevated urinary MMA [47], whereas in the above two included studies, initial folate and vitamin B12 status were in the normal range [32,51].

In conclusion, the available clinical studies show a potential role for probiotics to improve folate and vitamin B12 status. Of the three studies analyzed in this review, two studies yielded positive results. The clinical trials, however, were not consistent in strains, concentrations of probiotic bacteria, or duration of intervention.

#### 3.3.3. Probiotic Supplementation and Mineral Status

Three studies assessed the association between probiotic intake and calcium status in healthy pediatric [50], geriatric [48], and postmenopausal participants [16]. Two studies were cross-over double-blind randomized controlled trials [16,48] and one was a parallel randomized double-blind trial [50]. One study supplemented fermented milk with viable *L. helveticus* (at least 10^8^ CFU/mL) and *S. thermophilus* (dosage not mentioned) in geriatric subjects [48]. The initial calcium status for all subjects was in the normal range and remained within the normal range after four weeks of supplementation. The results indicated an improvement in serum calcium in the probiotic group compared to placebo. The randomization process, blinding, and concealment was not explicitly reported. There are also some concerns about the proportion of missing data, as it is not the same in the two groups (5 vs. 12 in probiotic and placebo groups, respectively). The second study involved postmenopausal women who were supplemented with milk fermented with *L. helveticus,* but no dosage for probiotic bacteria was reported [16]. The results showed an increasing trend in serum calcium after probiotic supplementation (one day intake, six days washout, crossover, one day intake) compared to control milk. There was no significant difference in serum ionised calcium, phosphate and urinary calcium levels between the two groups. There was some concern due to lack of explicit description of the randomization process. In another study, healthy pediatric individuals were supplemented with synbiotic tablets (*L. plantarum, L. acidophilus, B. infantis, B. lactis* (45 × 10^9^ CFU) and FOS) [50]. This latter study showed a 4% increase in serum calcium levels after synbiotic intake while, in the placebo group, there was 7% reduction in serum calcium; however, statistical significance was not indicated. The risk of bias assessment revealed some concerns as there is no explicit explanation of blinding. The last two studies did not report the initial calcium status of the subjects. In concert with the above studies, healthy pregnant women that were supplemented with a probiotic yogurt (*L. acidophilus* and *B. lactis*; 1 × 10^7^ CFU) reported a maintenance of serum calcium levels when compared with conventional yogurt [71]. Several mechanisms have been proposed regarding the effect of probiotics on serum calcium levels. The production of short-chain fatty acids (SCFAs) by probiotics can affect calcium absorption through a direct effect on cecal villi by increasing their surface area and, therefore, absorption [72]. SCFAs may also increase calcium-binding protein expression and paracellular calcium transport [72,73]. Moreover, higher levels of SCFAs following probiotic production can decrease the pH of the cecum and colon, enhancing calcium solubility and absorption [72,74]. The study by Narva et al. 2004 [16] suggests that *L. helveticus* could positively affect calcium metabolism by decreasing serum parathyroid hormone (PTH) and increasing serum calcium concentrations. The reduction in PTH levels may result from a facilitated calcium uptake by enterocytes after fermented milk consumption. One proposed mechanism is a reduction in the rate of gut emptying after fermented milk consumption. This reduction may occur due to an increased viscosity or decreased pH of fermented milk [75]. The other possible mechanism could be through the formation of caseinophosphopeptide [76], which inhibits the formation of insoluble calcium salt and increases the amount of soluble calcium [77,78]. These mechanisms of action need to be confirmed with further investigations [79,80,81]. In conclusion, two out of three studies have shown that probiotic consumption improves calcium status. In particular, it seems that *L. helveticus* may be beneficial in improving calcium levels.

Three studies assessed the association between probiotic intake and zinc status in healthy children [45,46,50]. Overall, 579 individuals were included in these three clinical trials. Two studies showed no significant change in zinc status after probiotic supplementation [46,50]. A randomized double-blind placebo-controlled trial compared the effect of low calcium and regular calcium milk with probiotic milk (regular calcium milk with *L. casei* 4311 (5 × 10^8^ CFU/d) and regular calcium milk with *L. reuteri* 17,938, (5 × 10^8^ CFU/d) [46]. Initial serum zinc levels showed that the subjects were zinc deficient, and 65 to 78% of subjects had zinc deficiency in each group, but probiotic intervention was ineffective in improving zinc status. This study revealed a low risk of bias. Another study was a randomized double-blind trial that evaluated the effect of a synbiotic containing *L. plantarum, L. acidophilus, B. infantis, B. Lacti,* prebiotic FOS, lactose free, gluten free, 45 × 10^9^ CFU in comparison to placebo [50]. The study showed that zinc levels increased by 1.7% in the treated group as compared to a decrease of about 3.5% in the placebo group, however, the mean initial zinc levels were not reported. The risk of bias assessment revealed some concerns as there is no explicit explanation of the randomization process and blinding. The third study showed that supplementation with a combination of zinc and 2.3 × 10^10^ CFU/g *L. plantarum* IS-10506 resulted in a significant increase in zinc levels after 90 days of supplementation, while probiotic supplementation in the absence of supplementary zinc did not change zinc status [45]. This trial revealed some concerns in the risk of bias assessment as there was not a complete explanation about the blinding and concealment process. At the beginning of the study, all subjects had normal zinc status. Beyond the differences in type, dose, and duration of interventions from the studies evaluating the influence of probiotics on zinc status, the combined intake of probiotics and zinc appears to be an important factor. Animal model studies suggested that the efficacy of probiotics may be increased by the combination of probiotics and synergistically acting prebiotic components [82] like FOS [83], phytins [84], maltodextrin KMS X-70 [85] working together with zinc [86]. Likewise, rat model studies have shown that the combination of probiotics and zinc can improve zinc bioavailability [87]. This may be due to a synergistic effect between the probiotic and zinc to improve histomorphology (villus height and total goblet cell count) of the intestine [88] and its integrity [89]. Therefore, co-supplementation of zinc and probiotics may amplify zinc absorption [45]. To conclude, data concerning the effect of probiotic intake on zinc status needs more investigation as the optimal strains, dose, and duration are still unknown. The three included studies were conducted solely on children, therefore, there is a need to evaluate the effect of probiotics on zinc levels in healthy adults and the elderly population.

Five of the 14 included studies in this review investigated the association between probiotic intake and iron status [28,46,48,49,52]. Two studies were randomized double-blind, placebo-controlled trials [46,49], two were non-randomized trials [28,52], and one was a cross-over double-blind randomized controlled study [48]. Several iron status-related outcomes were evaluated, including serum iron, ferritin, hemoglobin (Hb), hematocrit (Hct), total iron binding capacity (TIBC), transferrin, transferrin saturation, soluble transferrin receptor (sTfR) and hepcidin. Three studies examined serum iron levels [28,49,52], four studies examined ferritin [28,46,49,52], one study examined Hb [52], four studies examined both Hb and Hct [28,46,48,49], two studies examined sTfR [46,49], one study examined TIBC [52], and another one examined plasma transferrin, transferrin saturation, blood reticulocytes, mean reticulocyte hemoglobin and hepcidin [49]. A total of 721 healthy participants were included across these studies. Two studies were conducted on children [28,46], one on geriatric participants [48], one on females [52] and one on non-anemic female athletes with low iron stores [49]. Only one study, which was conducted on 20 female participants, indicated an 11% increase in serum iron levels after probiotic intake, however, the level of significance was not reported [52]. This trial was deemed to have a serious risk of bias as it was a non-randomized trial, and the authors did not elaborate on the confounders or ways to control them. The probiotic tested in this study was *L. plantarum,* in a dose of 1.1 × 10^9^ CFU, over seven consecutive days [52]. Axling et al. 2020 [49] also reported a trend (*p* = 0.0834) toward increased mean reticulocyte hemoglobin after *L. plantarum 299v* (10^10^ CFU/capsule) intake versus control. Moreover, borderline significant differences in ferritin levels following four weeks of *L. plantarum 299v* intake were observed (*p* = 0.056), which became significant when split into subgroups by baseline plasma ferritin levels; subgroups with baseline ferritin levels above 20 μg/L showed a significantly higher increase when compared to controls.

Clinical intervention trials have reported that non-heme iron absorption is increased after *L. plantarum* 299v consumption [13,24]. A recent meta-analysis of eight studies (including both above mentioned studies) suggested that *L. plantarum* increases the absorption of non-heme iron [90]. Several factors may lead to enhancement in iron absorption with probiotic treatment. A decreased pH due to the intake of probiotics that produce organic acids (e.g., lactic acid) after colonization in the proximal small intestine [91], or during transit through the gastrointestinal tract [13], may improve the solubility of the iron complex and increase iron absorption [13,92]. The increased acidity of the intestine, following probiotic organic acid production, can lead to the activation of phytases present in foods and may decrease the formation of insoluble iron complexes. Phytic acid found in food inhibits iron absorption due to the formation of insoluble complexes with iron at intestinal pH. Phytase activation may also improve iron absorption through a reduction in phytic acid levels [13]. Organic acids, produced during the fermentation process, slow gastric emptying, which may improve iron absorption [93] by increasing exposure of iron to the proximal intestinal epithelium [94,95]. Another proposed mechanism, demonstrated by *L. plantarum* in a human intestinal Caco-2/HT29 MTX cell model, is an increased level of a ferric reductase, duodenal cytochrome B, which positively influences iron absorption [96].

Subjects with normal iron status may respond differently to probiotic intervention than iron-deficient individuals. In the only clinical trial where serum iron was improved, initial ferritin status was not reported [52]. Three studies have been conducted on subjects with low ferritin [28,49] and Hb levels [48]. In one of the studies, the initial mean serum ferritin was normal [46]; however, 27–33% of subjects were iron-deficient in each group. It is expected that the participants with adequate baseline iron status may not experience significant improvements. Axling et al. 2020, however, showed that following *L. plantarum 299v* supplementation for four weeks, participants with a baseline ferritin level above 20 μg/L had higher ferritin levels compared to the control. Therefore, the initial iron status may impact the influence of probiotics on changes in iron status. In conclusion, the results of studies concerning the effect of probiotic intake on iron status are not congruent. The results are highly dependent on the strain, dose, and duration of probiotic supplementation as well as the initial iron status of the subjects. It seems that *Lactobacillus* species, notably *L. plantarum,* can be effective in improving iron status. Further investigations are needed to confirm these results.

### 3.4. Gaps in Current Research and Reasons for the Conflicting Results

The strain, dose, and duration of intervention are important to consider when evaluating the impact of probiotics on micronutrient status. After determining the most efficacious strains of probiotics for improving micronutrient status, there is a need to assess the strains at different doses to determine the ideal amount of CFUs to administer. There is no common recommended dose for all probiotics and the optimal dose may vary for different strains and the outcome under investigation. Some probiotic products are effective at 100 million CFU/day while others are recommended at over 1 trillion CFU/day [97]. In the present review, probiotic dosage in all included studies was more than 10^7^ CFU; however, two studies did not report probiotic dosage [16,47]: Elmadfa et al. 2001 did not mention the dosage of yogurt starter culture (*S. thermophilus* plus *L. acidophilus*) [42] and Gohel et al. 2016 did not mention the *S. thermophilus* dosage of milk fermented with *L. helveticus* and *S. thermophilus* [48]. The duration of probiotic interventions varies widely, and the optimal time needed for probiotic treatment is unknown. Intervention duration across the included studies ranged from 7 days to 6 months in parallel RCTs and nonrandomized clinical trials, which may contribute to the differences in reported results. More studies are needed to determine how long a specific probiotic will take to colonize, alter the microflora, and have an impact on specific indicators of (or factors that influence) micronutrient status.

It is important to consider that the effects of probiotics may be species-specific and may vary from one strain to another. Many strains or combinations of strains have been used in the included studies. There are suggestions of a possible effect of *L. acidophilus* [51] and VSL#3 [32] on folate and vitamin B12 status, *L. plantarum* on iron status [52] and *L. helveticus* on calcium status [16,48]. The most efficacious strains or combinations of strains for improving specific micronutrient status have yet to be identified. Moreover, in the studies that have used multi-species/strain probiotics, it is impossible to identify which strain may be exerting the beneficial effects. A previous study has indicated that probiotic mixtures may be more effective than a single strain probiotic [98]. This effect may be due to a synergistic interaction between strains or a consequence of the higher dose of bacteria used in multi-species/strain probiotics. Clearly, there is a need for more studies using individual strains to provide better insight regarding the most effective strains.

Beyond the differences in strains, dose, and duration of intervention, there may be several other factors such as baseline micronutrient status that contribute to the conflicting results regarding the effects of probiotics on micronutrient status in healthy subjects. First, of the three studies in which micronutrient status was a secondary outcome [32,46,50], two did not find significant effects of probiotic intake on calcium, zinc, iron and vitamins A and D status [46,50]. These studies likely did not have enough power to detect a significant effect of probiotic supplementation on micronutrient status. The study conducted by Agustina et al., 2013 had 494 participants and was underpowered (1–46%) to detect differences in iron and zinc status [46]. Ballini et al., 2019 had a small sample size (40 participants) and did not report the achieved power [50]. Adherence to the probiotic intake is another factor that contributes to the observed effects of the intervention on micronutrient status. Seven trials did not monitor adherence [16,42,43,44,50,51,52], and three studies did not report adherence rates [45,47,48]. If participants do not adhere to the probiotic intervention, observation of a true effect will be much less likely.

Probiotics may exert their beneficial effects through gut microbiota alteration. Changes in gut microbiota may occur through the ability of probiotics to suppress the growth of other microorganisms by the production of antimicrobial agents [99,100], their ability to compete with other intestinal bacteria for receptors on the intestinal mucosa [101], and/or increase the integrity of the intestinal barrier and decrease the translocation of bacteria [102]. Based on previous studies of animal models [103,104,105,106] and clinical trials [107,108], probiotics may balance the gut microflora; however, this effect of probiotics has not been observed in all human studies [109,110]. If probiotics consumption can lead to a change in gut bacteria composition and increase the relative abundance of micronutrient-producing bacteria, it may affect micronutrient status. However, further investigation into the ability of the gut microbiota to contribute to micronutrient status is required. In the present review, only one of the included studies assessed gut microbiota composition, but whether changes resulting from probiotic intake are beneficial is not clear [32]. An understanding of gut microbiome composition and microbial interactions in the gut is important to contextualize any change resulting from the probiotic intervention. Additionally, more research on the microbiota-host interplay is needed for a better understanding of the interaction of probiotics and nutrient intake.

Other issues that may impact probiotic effects on micronutrient status are related to the properties of the probiotic, such as acid and bile tolerance, adhesion properties and competitiveness against pathogens. The ability of probiotics to adhere to the intestinal mucosa allows the bacteria to exert their beneficial effects longer by increasing their time of persistence in the intestine [111,112]. Acid and bile tolerance are important for passage through the stomach and intestine. Acid tolerance is also important for the survival of probiotics in food carriers, such as yogurts and fermented milk, both of which have a relatively low-pH environment. These characteristics are strain dependent. Previous studies have shown that *L. casei* strains survive passage through the stomach and are able to colonize the colon [56]. Acid and bile tolerance, adhesion properties and competitiveness against pathogens for *L. plantarum* [113,114] and *L. helveticus* have also been proven previously [115,116,117,118]. However, only three of the included studies reported the properties of the probiotics used by citing previous work [42,45,48].

A number of other aspects require adequate monitoring and assessment in probiotic supplementation studies. For example, survival of probiotic bacteria in probiotic supplements and/or functional foods in terms of shelf-life and until consumption is essential to ensure that the probiotic dosage consumed is accurate. Monitoring of the total viable bacterial count is crucial. Only three of the included studies verified the viability of the probiotic bacteria [46,48,52]. Considering an appropriate washout period for the possible use of antibiotics in the inclusion criteria is necessary to avoid their effects on gut bacteria and ensuing confounding effects on the results. A growing number of studies have shown that antibiotics may affect gut microbiota through direct or indirect mechanisms (changes in total bacterial numbers and microbial diversity or changes in bacterial metabolites that affect the growth of other microbiota) [119]. Although antibiotics are administered to eliminate pathogenic bacteria, they may also kill non-pathogenic bacteria due to their broad-spectrum activity [120]. Of the included studies, five studies did not consider antibiotic washout in inclusion criteria before embarking in the study [16,28,47,49,52]. Adequate dietary assessment is also important together with consideration of baseline micronutrient status. Three out of fourteen included studies did not monitor the dietary intake of participants [45,49,52]. One study exhibited vague dietary control and only mentioned that participants will “keep a dietary protocol” [42], participants in another trial received standardized meals during study days [16], and in another study, participants received dietary advice with or without probiotics [32].

Overall, the results of this systematic review suggest that, although not all studies showed consistent results, probiotic consumption may influence vitamin B12, folate, calcium, zinc and iron status in healthy subjects. Heterogeneity of the studies, in terms of probiotic strain/species, administered form or doses, co-administration of the micronutrient measured, and the duration of intervention, limit the generalizability of these findings. Despite animal trial evidence regarding the beneficial effects of probiotics on the absorption of certain micronutrients, the effects of probiotics on micronutrient status still needs further study to fill several gaps in this field such as the ideal duration of treatment, dosage, and strain of probiotic to achieve efficacy.

## 4. Strengths and Limitations

The strengths of this systematic review were that: (a) this was conducted according to the standard PRISMA guidelines; (b) multiple databases were searched; and (c) the studies were considered on *a priori* inclusion/exclusion criteria. Additionally, two review authors screened the titles and extracted the data from the studies that were selected. The limitations of this review were: (a) the high heterogeneity of the selected studies in terms of the wide age range of the participants and the varying durations of treatments, particularly as longer treatment times might be more efficacious; (b) selected studies evaluated single or multispecies probiotics and synbiotics. When multispecies probiotics are used, it is not clear which species exerts the beneficial effect; (c) this review was based on the results of fourteen trials, which is not representative towards identifying all the possible micronutrient and health benefits associated with probiotic use; (d) The selected studies were limited to those that were published in English, therefore, there is a risk of publication bias; (e) most of the studies took place in European countries limiting the generalizability of the findings; (f) only one study had a low risk of bias; and (g) the number of studies per each individual probiotic strain and micronutrient association rarely exceeded one study.

## 5. Conclusions

This systematic review has provided an update on the effects of probiotic supplementation on micronutrient status and identified targeted future directions for research in the field. The review showed that despite the diversity of the published studies, the intake of probiotics in healthy subjects may result in improvements of vitamin B12, folate, calcium, zinc, and iron status. Additional well-designed double-blind randomized controlled trials are warranted to determine the most effective strains of probiotics and optimal doses and durations of intervention. In view of the complexities of inter-individual variability in terms of nutrient absorption, distribution, and metabolism as well as the metabolic activities of gut microbiota, further research is needed to study how such variations influence the impact of probiotics on micronutrient status.

## Supplementary Materials

The following are available online at https://www.mdpi.com/article/10.3390/nu13093001/s1, Figure S1: Bias assessment of parallel and cross-over RCTs. * cross-over studies. Green circles with plus sign = low risk of bias, yellow circle with minus sign = some concerns. Figure S2. Bias assessment of non-randomized clinical trials. Green circles with plus sign = low risk of bias, blue circle with question mark= no information, red circle with cross sign = serious risk of bias. Table S1: Studies that assessed the effect of probiotic supplementation on micronutrient status in healthy subjects.

## Appendix A

Detailed Search Strategy Including Full Electronic Search Strategy for PubMed, Scopus, and Embase.

### Appendix A.1. PubMed Search Strategies

(probiotics[MeSH] OR probiotic[tiab] OR *Lactobacillus*[tiab] OR *Saccharomyces*[tiab] OR *Bifidobacterium*[tiab] OR *Streptococcus*[tiab] OR *Enterococcus*[tiab] OR *Escherichia*[tiab] *Bacillus*[tiab] OR fermented foods and beverages[MeSH] OR fermented food[tiab] OR fermented beverages[tiab]).

*AND*

(micronutrient[Mesh] OR micronutrient[tiab] OR vitamin D[Mesh] OR vitamin D[tiab] OR ergocalciferol[tiab] OR cholecalciferol[tiab] OR vitamin A[MeSH] OR vitamin A[tiab] OR retinol[tiab] OR retinal[tiab] OR retinyl ester[tiab] OR all-trans-retinol[tiab] OR vitamin E[Mesh] OR vitamin E[tiab] OR tocopherol *[tiab] OR tocotrienol *[tiab] OR vitamin K[Mesh] OR vitamin K[tiab] OR phylloquinone[tiab] OR menaquinone[tiab] OR menadione[tiab] OR ascorbic acid[Mesh] OR ascorbic acid[tiab] OR vitamin C[tiab] OR thiamine[MeSH] OR thiamine[tiab] OR vitamin B1[tiab] OR riboflavin[MeSH] OR riboflavin[tiab] OR vitamin B2[tiab] OR niacin[Mesh] OR niacin[tiab] OR niacinamide[Mesh] OR niacinamide[tiab] OR vitamin B3[tiab] OR choline[MeSH] OR choline[tiab] OR phosphatidylcholine[MeSH] OR phosphatidylcholine[tiab] OR vitamin B6[MeSh] OR vitamin B6[tiab] OR pyridoxine[tiab] OR pyridoxal-5-phosphate[tiab] OR pyridoxamine[tiab] OR folic acid[MeSH] OR folic acid[tiab] OR vitamin B9[tiab] OR folate[tiab] OR vitamin B12[Mesh] OR vitamin B12[tiab] OR transcobalamin[Mesh] OR transcobalamin[tiab] OR hydroxocobalamin[tiab] OR pantothenic acid[MeSH] OR pantothenic acid[tiab] OR vitamin B5[tiab] OR biotin[MeSH] OR biotin[tiab] OR vitamin H[tiab] OR elements[MeSH] OR mineral[tiab] OR calcium[MeSH] OR calcium[tiab] OR Ca[tiab] OR iron, dietary[Mesh] OR dietary iron[tiab] OR Fe[tiab] OR zinc[MeSH] OR zinc[tiab] OR Zn[tiab] OR magnesium[MeSH] OR magnesium[tiab] OR Mg[tiab] OR chromium[MeSH] OR chromium[tiab] OR Cr[tiab] OR copper[MeSH] OR copper[tiab] OR Cu[tiab] OR potassium[MeSh] OR potassium[tiab] OR K[tiab] OR selenium[MeSh] OR selenium[tiab] OR Se[tiab] OR phosphorus, dietary[MeSH] OR phosphates[MeSh] OR phosphates[tiab] OR manganese[MeSH] OR manganese[tiab] OR Mn[tiab] OR molybdenum[MeSH] OR molybdenum[tiab] OR Mo[tiab] OR sodium[MeSH] OR sodium[tiab] OR Na[tiab] OR iodine[MeSH] OR iodine[tiab] OR fluorides[MeSH] OR fluorides[tiab] OR chlorides[MeSH] OR chlorides[tiab] OR Cl[tiab] OR carotenoids[MeSh] OR carotenoids[tiab] OR beta carotene[tiab] OR zeta carotene[tiab] OR zeaxanthins[tiab] OR lutein[tiab] OR alpha-carotene[Supplementary Concept] OR alpha-carotene[tiab] OR carotene[tiab] OR carotenes[tiab] OR beta-cryptoxanthin[tiab] OR lycopene[tiab] OR astaxanthin[tiab] OR xanthophyll[tiab]).

*AND*

(Human[MeSH] OR Human[tiab] OR adult *[tiab] OR child *[tiab] OR elder *[tiab] OR men[tiab] OR women[tiab]).

*AND*

(randomized controlled trials as topic [Mesh] OR controlled clinical trial [Publication Type] OR random *[tiab] OR intervention[tiab] OR clinical [tiab] OR trial [tiab] OR placebo[tiab] OR double-blind[tiab] OR single-blind[tiab].

*AND*

(healthy volunteers [MeSH] OR healthy [tiab]).

### Appendix A.2. Scopus Search Strategy

((TITLE-ABS-KEY (probiotic *)OR TITLE-ABS-KEY (Lactobacillus)OR TITLE-ABS-KEY (Saccharomyces)OR TITLE-ABS-KEY (Bifidobacterium)OR TITLE-ABS-KEY (Streptococcus)OR TITLE-ABS-KEY (Enterococcus)OR TITLE-ABS-KEY (Escherichia)OR TITLE-ABS-KEY (Bacillus)OR TITLE-ABS-KEY (“Fermented food *”)OR TITLE-ABS-KEY (“Fermented beverages *”))).

*AND*

((TITLE-ABS-KEY (micronutrients *)OR TITLE-ABS-KEY (vitamin *)OR TITLE-ABS-KEY (ergocalciferols)OR TITLE-ABS-KEY (cholecalciferol)OR TITLE-ABS-KEY (retinol)OR TITLE-ABS-KEY (retinal)OR TITLE-ABS-KEY (“retinyl ester”)OR TITLE-ABS-KEY (“all-trans-Retinol”)OR TITLE-ABS-KEY (tocopherol *)OR TITLE-ABS-KEY (tocotrienol *)OR TITLE-ABS-KEY (phylloquinone)OR TITLE-ABS-KEY (menaquinone)OR TITLE-ABS-KEY (menadione)OR TITLE-ABS-KEY (“ascorbic acid”)OR TITLE-ABS-KEY (thiamine)OR TITLE-ABS-KEY (riboflavin)OR TITLE-ABS-KEY (niacin)OR TITLE-ABS-KEY (niacinamide)OR TITLE-ABS-KEY (choline)OR TITLE-ABS-KEY (phosphatidylcholine)OR TITLE-ABS-KEY (pyridoxine)OR TITLE-ABS-KEY (“pyridoxal-5-phosphate”)OR TITLE-ABS-KEY (pyridoxamine)OR TITLE-ABS-KEY (“folic acid”)OR TITLE-ABS-KEY (folate)OR TITLE-ABS-KEY (transcobalamins)OR TITLE-ABS-KEY (hydroxocobalamin)OR TITLE-ABS-KEY (“pantothenic acid”)OR TITLE-ABS-KEY (biotin)OR TITLE-ABS-KEY (element *)OR TITLE-ABS-KEY (mineral *)OR TITLE-ABS-KEY (calcium)OR TITLE-ABS-KEY (iron)OR TITLE-ABS-KEY (zinc)OR TITLE-ABS-KEY (magnesium)OR TITLE-ABS-KEY (chromium)OR TITLE-ABS-KEY (copper)OR TITLE-ABS-KEY (potassium)OR TITLE-ABS-KEY (selenium)OR TITLE-ABS-KEY (phosphorus)OR TITLE-ABS-KEY (phosphate *)OR TITLE-ABS-KEY (manganese)OR TITLE-ABS-KEY (molybdenum)OR TITLE-ABS-KEY (sodium)OR TITLE-ABS-KEY (iodine)OR TITLE-ABS-KEY (fluorides)OR TITLE-ABS-KEY (chlorides)OR TITLE-ABS-KEY (carotene *)OR TITLE-ABS-KEY (“beta Carotene”)OR TITLE-ABS-KEY (“zeta carotene”)OR TITLE-ABS-KEY (zeaxanthins)OR TITLE-ABS-KEY (lutein)OR TITLE-ABS-KEY (“alpha carotene”)OR TITLE-ABS-KEY (“Beta cryptoxanthin”)OR TITLE-ABS-KEY (lycopene)OR TITLE-ABS-KEY (astaxanthin)OR TITLE-ABS-KEY (xanthophyll))).

*AND*

((TITLE-ABS-KEY (“randomized Controlled Trials”)OR TITLE-ABS-KEY (“controlled clinical trial”)OR TITLE-ABS-KEY (random *)OR TITLE-ABS-KEY (intervention)OR TITLE-ABS-KEY (clinical)OR TITLE-ABS-KEY (trial)OR TITLE-ABS-KEY (placebo)OR TITLE-ABS-KEY (“double-blind”)OR TITLE-ABS-KEY (“single-blind”))).

*AND*

((TITLE-ABS-KEY (“healthy volunteers”)OR TITLE-ABS-KEY (“healthy adult”)OR TITLE-ABS-KEY (“healthy children”)OR TITLE-ABS-KEY (“healthy subject”)OR TITLE-ABS-KEY (“healthy participant”)OR TITLE-ABS-KEY (healthy))).

*AND*

((TITLE-ABS-KEY (Human)OR TITLE-ABS-KEY (adult *)OR TITLE-ABS-KEY (child *)OR TITLE-ABS-KEY (men)OR TITLE-ABS-KEY (women)).

### Appendix A.3. Embase Search Strategy

(probiotic *: ab,ti OR *Lactobacillus*:ab,ti OR *Saccharomyces*:ab,ti OR *Bifidobacterium*:ab,ti OR *Streptococcus*:ab,ti OR *Enterococcus*:ab,ti OR *Escherichia*:ab,ti OR Bacillus:ab,ti OR “fermented food *”:ab,ti OR “fermented beverages *”:ab,ti).

*AND*

(micronutrients *:ab,ti OR vitamin *:ab,ti OR ergocalciferols:ab,ti OR cholecalciferol:ab,ti OR retinol:ab,ti OR retinal:ab,ti OR “retinyl ester”:ab,ti OR “all-trans-Retinol”:ab,ti OR tocopherol *:ab,ti OR tocotrienol *:ab,ti OR phylloquinone:ab,ti OR menaquinone:ab,ti OR menadione:ab,ti OR “ascorbic acid”:ab,ti OR thiamine:ab,ti OR riboflavin:ab,ti OR niacin:ab,ti OR niacinamide:ab,ti OR choline:ab,ti OR phosphatidylcholine:ab,ti OR pyridoxine:ab,ti OR “pyridoxal-5-phosphate”:ab,ti OR pyridoxamine:ab,ti OR “folic acid”:ab,ti OR folate:ab,ti OR transcobalamins:ab,ti OR hydroxocobalamin:ab,ti OR “pantothenic acid”:ab,ti OR biotin:ab,ti OR element *:ab,ti OR mineral *:ab,ti OR calcium:ab,ti OR iron:ab,ti OR zinc:ab,ti OR magnesium:ab,ti OR chromium:ab,ti OR copper:ab,ti OR potassium:ab,ti OR selenium:ab,ti OR phosphorus:ab,ti OR Phosphate *:ab,ti OR manganese:ab,ti OR molybdenum:ab,ti OR sodium:ab,ti OR iodine:ab,ti OR fluorides:ab,ti OR chlorides:ab,ti OR carotene *:ab,ti OR “beta Carotene”:ab,ti OR “zeta carotene”:ab,ti OR zeaxanthins:ab,ti OR lutein:ab,ti OR “alpha carotene”:ab,ti OR “Beta cryptoxanthin”:ab,ti OR lycopene:ab,ti OR astaxanthin:ab,ti OR xanthophyll:ab,ti).

*AND*

(Human OR adult *:ab,ti OR child *:ab,ti OR elder *:ab,ti OR men:ab,ti OR women:ab,ti).

*AND*

(randomized controlled trials:ab,ti OR controlled clinical trial:ab,ti OR random *:ab,ti OR intervention:ab,ti OR clinical:ab,ti OR trial:ab,ti OR placebo:ab,ti OR double-blind:ab,ti OR Single-blind:ab,ti).

*AND*

(“healthy volunteers”:ab,ti OR healthy:ab,ti).

The appendix is an optional section that can contain details and data supplemental to the main text—for example, explanations of experimental details that would disrupt the flow of the main text but nonetheless remain crucial to understanding and reproducing the research shown; figures of replicates for experiments of which representative data is shown in the main text can be added here if brief, or as supplementary data. Mathematical proofs of results not central to the paper can be added as an appendix.
