# Supplementary material for: Probiotic Supplementation and Micronutrient Status in Healthy Subjects: A Systematic Review of Clinical Trials
Source: Nutrients. 2021 Aug 28;13(9):3001. doi: 10.3390/nu13093001 (PMC8472411; doi:10.3390/nu13093001)
Supplement: Supplementary file 1 [file nutrients-13-03001-s001.zip › Figure S1.pdf]

Supplementary Documents:

Figure S1. Bias assessment of parallel and cross-over RCTs. * cross-over studies. Green circles with plus sign = low risk of bias, yellow circle with minus sign = some concerns.

Study: Author/ year	Bias arising from randomization process	Bias due to deviation from intended intervention	Bias due to missing outcome data	Bias in measurement of the outcome	Bias in selection of the reported result	Overall result
Agustina R et al., 2013	+	+	+	+	+	+
Axling U et al., 2020	+	-	+	+	+	-
Ballini A et al., 2019	+	-	+	+	-	-
Donaldson M S et al., 2000	-	-	+	+	+	-
Fabian E and Elmadfa I., 2007	-	-	+	+	+	-
Fabian E et al., 2008	-	-	+	+	+	-
Mohammad A et al., 2006	-	-	+	+	+	-
Surono I.S et al., 2014	+	-	+	+	+	-
Valentini L et al., 2015	+	-	+	+	+	-
*Gohel M et al., 2016	-	-	-	+	+	-
*Narva M et al., 2004	-	+	+	+	+	-
