# Supplementary material for: Probiotic Supplementation and Micronutrient Status in Healthy Subjects: A Systematic Review of Clinical Trials
Source: Nutrients. 2021 Aug 28;13(9):3001. doi: 10.3390/nu13093001 (PMC8472411; doi:10.3390/nu13093001)
Supplement: Supplementary file 1 [file nutrients-13-03001-s001.zip › Figure S2.pdf]

Figure S2. Bias assessment of non-randomized clinical trials. Green circles with plus sign = low risk of bias, blue circle with question mark= no information, red circle with cross sign = serious risk of bias.

Study: Author/ year	Bias due to confounding	Bias due to selection of participants	Bias in classification of interventions	Bias due to deviations from intended interventions	Bias due to missing data	Bias in measurement of outcomes	Bias in selection of the reported result	Overall result
Elmadfa I et al., 2001	X	+	+	+	+	+	+	X
Silva M et al., 2008	X	+	+	X	X	+	X	X
Korčok D et al., 2018	X	+	+	+	?	+	+	X
