# Supplementary material for: Probiotic Supplementation and Micronutrient Status in Healthy Subjects: A Systematic Review of Clinical Trials
Source: Nutrients. 2021 Aug 28;13(9):3001. doi: 10.3390/nu13093001 (PMC8472411; doi:10.3390/nu13093001)
Supplement: Supplementary file 1 [file nutrients-13-03001-s001.zip › supplement-table.pdf]

Table S1. Studies that assessed the effect of probiotic supplementation on micronutrient status in healthy subjects.

Reference	Study Design	Population group	Intervention	Comparator	Dose	Format	Main Results
Agustina et al. 2013 [46]	RDBPC	494 healthy children (aged 1–6 years, non-breastfed)	Intervention Group 1: regular calcium milk with *L. casei* 4311<br><br>Intervention Group 2: Regular calcium milk with *L. reuteri* 17,938	Placebo Group 1: low calcium milk<br><br>Placebo Group 2: regular calcium milk	180 mL<br>Twice a day for 6 months<br>Group 1: *L. casei* 4311, 5×10^8 CFU/d<br>Group 2: *L. reuteri* 17,938 , 5×10^8 CFU/d	Milk and coated straws	Significant decrease in Hb, Hct and serum ferritin in all groups from baseline but no significant difference between PROB and PC<br><br>Significant increase in sTfR in all groups from baseline but no significant difference between PROB and PC<br><br>No significant changes in serum zinc
Axling et al. 2021 [49]	RDBPC	39 healthy non-anemic female athletes with low iron stores, (aged 16–40 years)	*L. plantarum* 299v	Placebo: capsule with no probiotic	Daily one, for 12 weeks<br>*L. plantarum* 299v, 10^{10} CFU/capsule	Capsule	Significant increase in plasma ferrtin in both groups from baseline to 12 weeks but no significant difference between PROB and PC<br><br>Borderline significant difference in serum ferritin after 4 weeks between PROB and PC (p=0.056)<br><br>No significant results in mean reticulocyte hemoglobin, Hb, plasma iron, plasma transferrin, plasma transferrin saturation, EVF, blood reticulocytes, sTfR, hepcidin

Ballini et al. 2019 [50]	RDBCT	40 children<br>(aged 14–18 years)	*L. plantarum*, *L. acidophilus*, *B. infantis*, *B. Lactis*, combined with prebiotic fructo-oligosaccharides	Placebo: tablet with no probiotic	Daily one for 10 weeks<br>*L. plantarum*, *L. acidophilus*, *B. infantis*, *B. Lactis*, 45 × 10^9 CFU	Tablet	a<br>Increase in vitamin A in both groups from baseline<br><br>Vitamin D, calcium and zinc increased in synbiotic group but decreased in PC<br>(p values not reported)
Donaldson et al. 2000 [47]	RCT	24 vegan subjects<br>(aged 55 ± 9 years)	Group 2: 1 tablespoon of nutritional yeast daily<br>Group 3: Probiotic Formula (*L. plantarum, L. salivarius, L. acidophilus, B. bifidus* and *Bacillus subtilis)*<br>Group 4: Flora Food *(L. salivarius* and *L. plantarum*)	Group1: Placebo: sublingual cyanocobalamin tablet with no probiotic	Twice a day for 3 months<br>Group1: 500 µg for 3 days a week<br>Group 2: ~5 µg cyanocobalamin<br>Group 3 and 4: no dosage information reported	Capsule	Significant decrease in MMA in groups 1 and 2 from baseline but no significant change in groups 3 and 4
Elmadfa et al. 2001 [42]	CT	12 healthy adults<br>(aged 25–36 years)	*S. thermophilus* plus *L. acidophilus* and *L. casei* (*rhamnosus*)S GG (LGG)	Placebo:<br>Yogurt with thermally inactivated cultures	500 g daily for one month<br>*S. thermophilus* plus *L. acidophilus* and *L. casei* (*rhamnosus*)S GG (LGG), 5 ×10^7 CFU/g	Yogurt	Blood plasma:<br>After the first two weeks:<br>Significant decrease in plasma thiamin from baseline<br>Significant increase in FAD from baseline<br>No significant change in FMN, free riboflavin or P-5-P<br><br>After the second two weeks:<br>Significant decrease in plasma thiamin and FAD from baseline<br>No significant changes in FMN, free riboflavin or P-5-P

							Urine:<br>Significant decrease in pyridoxic acid<br>No significant changes in thiamin or B2 vitamers<br><br>Feces:<br>No significant changes in thiamin, B2 vitamers or pyridoxical and pyridoxic acid
Fabian and Elmadfa 2007 [43]	RCT	32 healthy women (aged 22–29 years)	Intervention group: probiotic yogurt: *S. thermophilus* and *L. bulgaricus*, enriched with the probiotic culture *L. paracasei* (subsp. *Paracasei*) (*L. casei* DN-114 001	Placebo: conventional Yogurt: *S. thermophilus, L. bulgaricus*	Daily for one month<br>First two weeks 100 g, second two weeks 200 g<br>intervention group: *S. thermophiles,* 2 × 10^8 CFU/g and *L. bulgaricus,* 10^7 CFU/g, enriched with the probiotic culture *L. paracasei* (subsp. *Paracasei*) (*L. casei* DN-114 001), 3.6 × 10^8 CFU/g<br>Placebo: *S. thermophilus,* 3.9 × 10^7 CFU/g, *L. bulgaricus,* 6.4 × 10^7 CFU/g	Yogurt	During continued yogurt intake (T1-T3), in both groups<br><br>Significant decrease in vitamin E, lycopene, zeaxanthin in both groups from baseline<br><br>Significant decrease in lutein and β-carotene from baseline only in PROB
Fabian et al. 2008 [44]	RCT	32 healthy women (aged 22-29 years)	Intervention group: probiotic yogurt, *S. thermophilus* + *L. bulgaricus* + *L. paracasei* (subsp. *Paracasei*) + *L. casei* DN-114 001	Placebo : conventional yogurt: *S. thermophiles*, *L. bulgaricus*	Daily for one month<br>First two weeks 100 g, second two weeks 200 g<br>intervention group: *S. thermophiles,* 2 × 10^8 CFU/g and *L. bulgaricus,* 10^7 CFU/g, enriched with the probiotic culture *L. paracasei* (subsp. *Paracasei*) (*L. casei* DN-114 001), 3.6 × 10^8 CFU/g<br>Placebo: *S. thermophilus,* 3.9 × 10^7 CFU/g, *L. bulgaricus,* 6.4 × 10^7 CFU/g	Yogurt	Blood:<br>After first two weeks:<br>No significant change in thiamin and P-5-P in both groups from baseline<br>Significant decrease in FAD in both groups from baseline<br>Significant increase in FMN and free riboflavin in both groups from baseline<br><br>After the second two weeks in both groups:

							Significant increase in thiamin, FMN and free riboflavin in both groups<br>Significant decrease in FAD in both groups<br>No significant changes in P-5-P in both groups<br><br>Urine:<br>No significant changes in thiamin, riboflavin, α-EGR, pyridoxic acid and α- EGOT
Gohel et al. 2016 [48]	RDBPC	59 healthy older people, (aged 64-74 years)	Fermented milk with *L. helveticus* MTCC 5463 and *S. thermophilus* MTCC 5460.	Placebo: feremented milk without probiotic	200 mL/day,<br>4 week intake, 4 week washout, crossover, 4 week intake and 4 week washout<br>At least 10^8 CFU/mL of viable *L. helveticus* and *S. thermophilus* MTCC 5460.	Milk	Significant increase in serum calcium in PROB and significant decrease in PC from baseline<br><br>Significant increase in Hct from baseline only in PROB<br><br>No significant change in Hb
Korčok et al. 2018 [52]	CT	20 healthy females, (aged 20 - 40 years)	*L. plantarum* 299v	Placebo: capsule with no probiotic	Daily one, for 7 days<br>*L. plantarum* 299v, 1.1 × 10^9 CFU	Capsule	a<br>An improvement in iron status in PROB compared to PC<br><br>No change in ferritin, TIBC and Hb<br>(p values not reported)
Mohammad et al. 2006 [51]	RCT	24 healthy children, (aged 11 years)	*L. acidophilus* (La1)	Placebo: yogurt without probiotic	2 cups yogurt daily for 42 days<br>*L. acidophilus* (La1), 5 x 10^9 CFU	Yogurt	Blood:<br>Significant increase in vitamin B12, folate only in PROB from baseline<br>Significant decrease in t-Hcy only in PROB from baseline<br>Urine:

						Significant decrease in MMA and t-Hcy only in PROB compared to baseline
Narva et al. 2004 [16]	RDBPC	20 healthy postmenopausal women (aged 50-78 years)	Fermented milk *with L. helveticus* LBK-16H bacteria and peptide concentrations of 14.5 mg/100 g of IPP and VPP together	Control milk fermented with *Lactococcus sp.* mixed culture	220 mL *L.helveticus* milk, and 420 mL control milk,<br>One day intake, 6 days washout, crossover, one day intake<br>no probiotic dosage information reported	Milk	Significant improvement in serum calcium in PROB compared to PC<br>No significant result in serum ionised calcium, phosphate and urinary calcium
Silva et al. 2008 [28]	CT	109 children,<br>(aged 2-5 years)	Probiotic group: same as placebo group + *L. acidophilus*	Placebo: whole milk with *S.thermophilus* + *L. bulgaricus* (0.8), culture of probiotic *L. acidophilus* (1.2)	80 mL daily for 101 days<br>*L. acidophilus* 10^8 CFU/mL	Milk	Significant decrease in serum ferritin in PROB, and significant increase in PC from baseline<br>Significant decrease in Hb in both groups but no significant difference between PROB and PC<br>Significant decrease in Hct in both groups from baseline<br>Significant decrease in serum iron from baseline only in PROB
Surono et al. 2014 [45]	RDBPCT	45 healthy infants<br>(aged 12-24 months)	Group 1: probiotic *L. plantarum* IS-10506,<br>Group 2: combination of probiotic and zinc	Group 3: zinc<br>Group 4: placebo	Daily for 90 days<br>Group 1: *L. plantarum* IS-10506, 2.3 × 10^{10} CFUs/g powder<br>Group 3: 20 mg zinc sulfate monohydrate	Powder	Significant improvement in serum zinc in groups 2 and 3 from baseline<br>Significant increase in serum zinc in group 2 compared to PC
Valentini et al. 2015 [32]	RCT	62 healthy older people,<br>(aged 65- 85 years)	Web-based dietary advice (RISTOMED platform) with supplementation of VSL#3 consisting of the following strains: *B. infantis* DSM 24737, *B. longum* DSM 24736, *B. breve* DSM 24732, *L. acidophilus* DSM 24735, *L.*	Web-based dietary advice (RISTOMED platform) alone	Twice a day for 8 weeks<br>112 billion lyophilized bacteria	Capsules	Significant improvement in vitamin B12, folate in PROB from baseline<br>Significant decrease in Hcy in PROB from baseline

		delbrückii ssp. bulgaricus DSM 24734, *L. paracasei* DSM 24733, *L. plantarum* DSM 24730, and *S. thermophilus* DSM 24731, in defined ratio)	

[a], signifies that p value or level of significance not reported; RDBPC, randomized double blind placebo control; RCT, randomized control trial; CT, clinical trial; PROB, probiotic; PC, placebo; Hb: hemoglobin; Hct: hematocrit; sTfR: soluble transferrin receptor; TIBC: Total iron binding capacity; FAD: Flavin adenine dinucleotide; FMN: Flavin mononucleotide; P-5-P: *pyridoxal 5'-phosphate;* EVF: Erythrocyte volume fraction; Hcy: Homocysteine; MMA: methylmalonic acid; α-EGR: erythrocyte glutathione reductase; α- EGOT; erythrocyte glutamate oxaloacetate transaminase.
